# Future Research and Clinical Directions in the Field of Men’s Mental Health: The Madrid Declaration

**DOI:** 10.3389/fpubh.2014.00242

**Published:** 2014-11-19

**Authors:** Leo Sher, Zoltan Rihmer, Javier Didia-Attas, Jose de Leon, Shih-Ku Lin, Carlos Roncero, Nestor Szerman, Timothy Rice

**Affiliations:** ^1^Icahn School of Medicine at Mount Sinai, New York, NY, USA; ^2^James J. Peters Veterans’ Administration Medical Center, Bronx, NY, USA; ^3^Semmelweis University, Budapest, Hungary; ^4^Hospital Italiano, Buenos Aires, Argentina; ^5^University of Kentucky, Lexington, KY, USA; ^6^Taipei Medical University, Taipei, Taiwan; ^7^Psychiatric Center, Taipei City Hospital, Taipei, Taiwan; ^8^University Hospital Vall Hebron, Universidad Autónoma de Barcelona, Barcelona, Spain; ^9^Complutense University of Madrid, Madrid, Spain

**Keywords:** mental disorders, men’s mental health, alcoholism, substance-related disorders, stress, psychological

The members of the World Federation of Biological Psychiatry’s Task Force on Men’s Mental Health met in Madrid in September 2014 to discuss the research and clinical directions in the field of Men’s Mental Health. Leo Sher, M.D. (USA), Zoltan Rihmer, M.D., Ph.D. (Hungary), Javier Didia-Attas, M.D. (Argentina), Jose de Leon, M.D. (USA), Shih-Ku Lin, M.D. (Taiwan), Carlos Roncero, M.D. (Spain), and Nestor Szerman, M.D. (Spain) participated in the meeting. The following consensus recommendations were made.

## Help-Seeking Behavior

Men seek help less frequently than women (1, 2). Young men in particular receive less health care and are more often uninsured (3). Without routine preventative care, risk factors, and warning signs for mental health concerns can go unobserved and unaddressed. Among men, and young men, in particular, masculinity is directly associated with receiving less healthcare and particularly mental health services (1, 4, 5). Research, clinical work, and public health interventions targeting misconceptions among males that a lifestyle involving poor self-care and lack of help seeking is masculine are needed.

## Impulsivity

Men are more impulsive and aggressive than women (6, 7). This leads to suicidality (6–8), substance abuse (9), and aggressive violence and homicidality (7, 10). There are defined neural correlates for this association (11). The focus of research and clinical work must be how to continue to further define these neural correlates, how to diagnose and treat impulsivity, and how to reduce access to lethal means.

## Alcohol and Drug Abuse in Men

There is a consistent and greater male use of alcohol, benzodiazepines, and illegal drugs of abuse (2, 12). This is particularly true with alcohol, especially in select cultures, including those of Eastern Europe and the Americas. Patterns of use and behavior additionally diverge. Research and clinical work should be focused on targeted interventions to reduce male substance abuse and its associated disruptive behavior, suicidality, and aggression.

## Work Related Stress

Employment and income expectations and concerns are traditionally of more importance to men (2, 13, 14). In Western cultures, men are expected to provide for their families. For men, it is more difficult to lose a social and economic status than for women. The widespread youth unemployment in many countries worldwide may have particularly negative impact upon young men (15). This needs to be studied in the context of stress reduction to prevent depression and suicide and to preserve function to help unemployed men to find new jobs.

## Bereavement

The process of bereavement after widowhood may be more difficult for husbands (16, 17), though recent data suggests many complex factors may underlie earlier evidence that supported this claim (18). As researchers and clinicians, we must explore the particular factors, which may make bereavement more difficult, and we must ask what kind of psychotherapy can be performed that may specifically target male vulnerability to complications of bereavement.

## Hormones

Testosterone levels in adults have been directly linked to impulsivity, aggression, desire to dominate, depression, and criminality (19, 20). Testosterone may produce this effect via organizational and activational effects. Organizational effects refer to the effects which testosterone produces on the developing central nervous system during pregnancy and adolescence, whereas activational effects refer to those which serum testosterone levels directly produce. Clinical and research work must define and develop the understanding of both these pathways.

## Pharmacokinetics

Male and female sex hormones exert divergent effects upon the cytochrome P450 system and other metabolic enzymes (21, 22). Female hormones are inhibitors of CYP1A2, and smoking, a CYP1A2 inducer, is usually more frequent in men. Males, thus, on average, have greater CYP1A2 activity than females and may need higher doses of some psychiatric medications, including clozapine. There is more need for research in these gendered pharmacokinetic differences that, once well-established, need to be incorporated by clinicians in their work.

## Social Awareness

There should be an increased public health awareness of differences in behavior and mental health between men and women. Public health interventions including education and primary preventative efforts focused on men are needed.

Men’s mental health needs more attention from clinicians, researchers, and health policy makers. This declaration serves as a preliminary communication of the World Federation of Biological Psychiatry’s Task Force on Men’s Mental Health concerning future directions for research, clinical care, and public health policy.

## Conflict of Interest Statement

The authors declare that the research was conducted in the absence of any commercial or financial relationships that could be construed as a potential conflict of interest.
